# 1,3′-Dimethyl-2-oxo-6′-phenyl-7′*H*-spiro­[indoline-3,4′-isoxazolo[5,4-*b*]pyridine]-5′-carbo­nitrile

**DOI:** 10.1107/S2414314626002324

**Published:** 2026-03-11

**Authors:** S. Logalakshmi, Panneerselvam Yuvaraj, Dhruba Jyoti Boruah, R. Raja, G. Puthilibai

**Affiliations:** a Department of Chemistry, Sri Sairam Engineering College (Autonomous), Chennai - 600 044, India; b Chemical Sciences & Technology Division CSIR-North East Institute of Science & Technology (NEIST) Assam - 785006 India; c Academy of Scientific and Innovative Research (AcSIR) Ghaziabad - 201002 India; d Department of Physics, Thiruthangal Nadar College, Chennai - 600 051, India; Katholieke Universiteit Leuven, Belgium

**Keywords:** crystal structure, indoline, pyridine, isoxazole, inter­molecular hydrogen bonds, heterocycle, π–π stacking

## Abstract

The title compound, C_22_H_16_N_4_O_2_, crystallizes in the monoclinic space group *P*2_1_/*c* with two independent mol­ecules, *A* and *B*, in the asymmetric unit. The isoxazole ring is inclined to the pyridine ring system, the indoline ring, and the phenyl ring by 8.00 (10), 70.93 (10), 35.89 (12), respectively, ° for mol­ecule *A*,and 4.24 (10), 84.62 (9),30.02 (11)° for mol­ecule *B*. In the crystal, mol­ecules are linked by C—H⋯O and C—H⋯N inter­actions, forming layers parallel to the (100) plane. In addition π–π inter­actions with centroid-to-centroid distances of 3.8057 (12) Å and a series of C—H⋯π inter­actions help to consolidate the packing in a three-dimensional architecture within the crystal. Additional electron density was localized in voids (398 Å^3^ solvent accessible volume) summing up to 92 electrons, which corresponds to approximately 3.5 mol­ecules of ethanol. The given chemical formula and other crystal data do not take into account the solvent.

## Abstract 2

In the two mol­ecules in the asymmetric unit of the title compound, C_22_H_16_N_4_O_2_, the isoxazole ring is inclined to the pyridine ring system, the indoline ring, and the phenyl ring by 8.00 (10), 70.93 (10), and 35.89 (12)°, respectively, for mol­ecule *A*, and 4.24 (10), 84.62 (9), and 30.02 (11)° for mol­ecule *B*. In the crystal, mol­ecules are linked by N—H⋯O, C—H⋯O and C—H⋯N inter­actions, forming layers parallel to the (100) plane.

## Graphical abstract

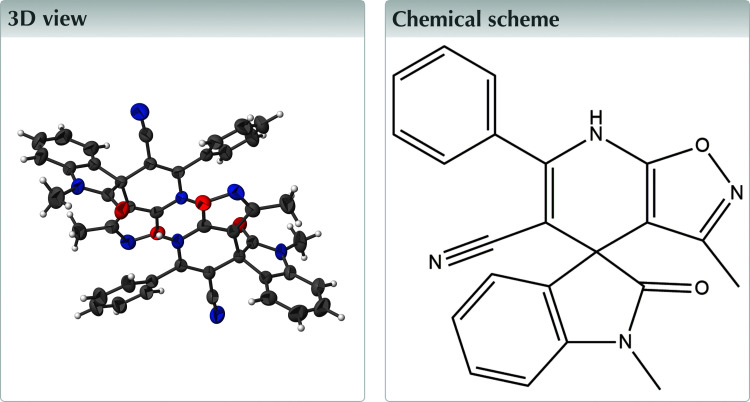

## Structure description

Nitro­gen-containing heterocyclic compounds have attracted the attention of many researchers during the decades-long historical development of organic synthesis (Dömling *et al.*, 2000 ▸). Many heterocyclic compounds exhibit many biological properties such as anti­convulsant, anti­tumour, anti­neoplastic, anti­septic, anti­viral, and hypnotic properties, *etc*. In particular, the indole nucleus occurs in a variety of natural products and medicinal agents (de Graaff *et al.*, 2012 ▸). Therefore, natural products with a heterocyclic ring structure are attracting considerable attention in the fields of pharmaceuticals and synthetic organic chemistry (Houlihan *et al.*,1992 ▸).

**Figure 1:**
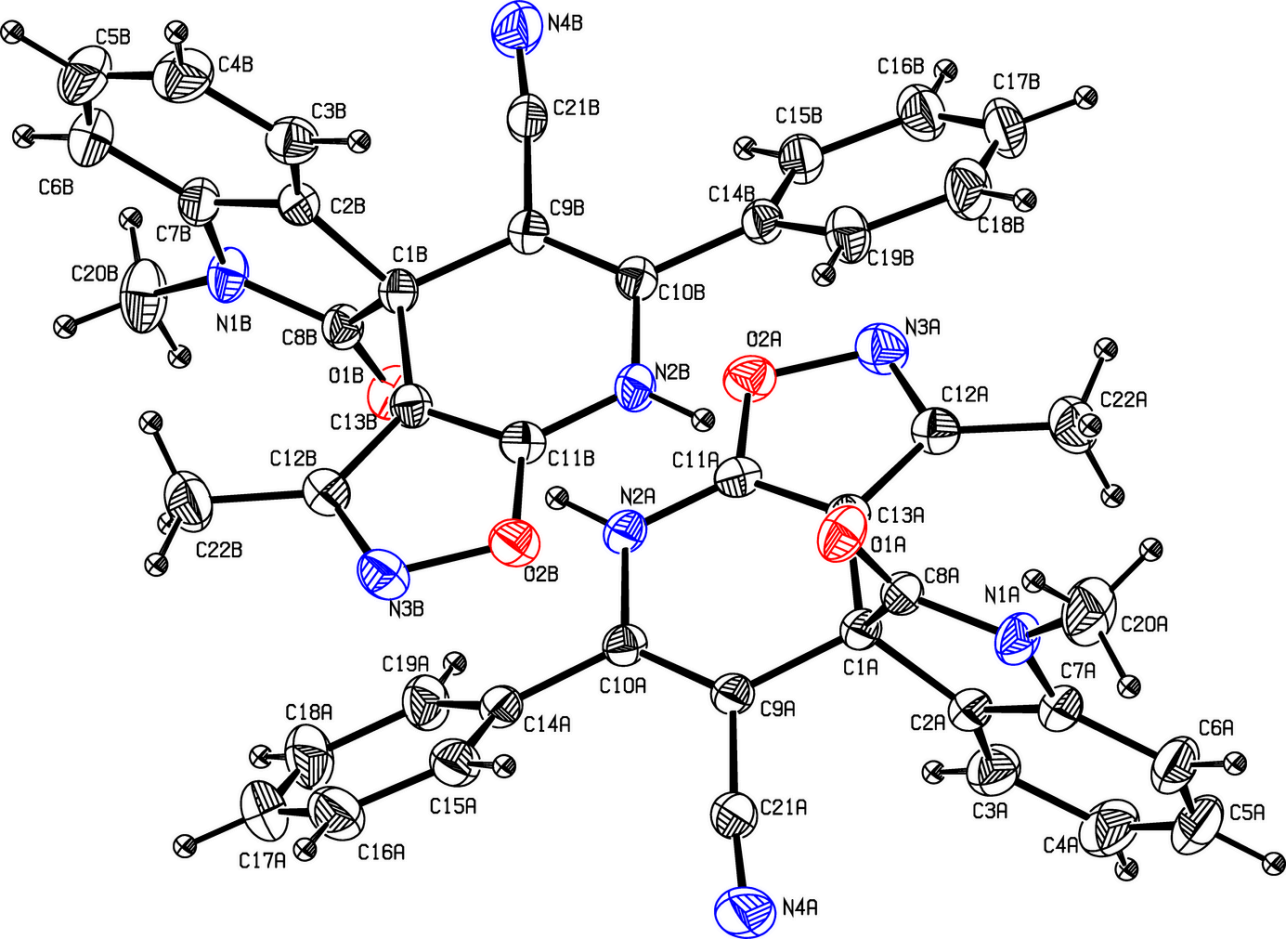
The mol­ecular structures of mol­ecules *A* and *B* in the title compound with displacement ellipsoids drawn at the 30% probability level.

**Figure 2:**
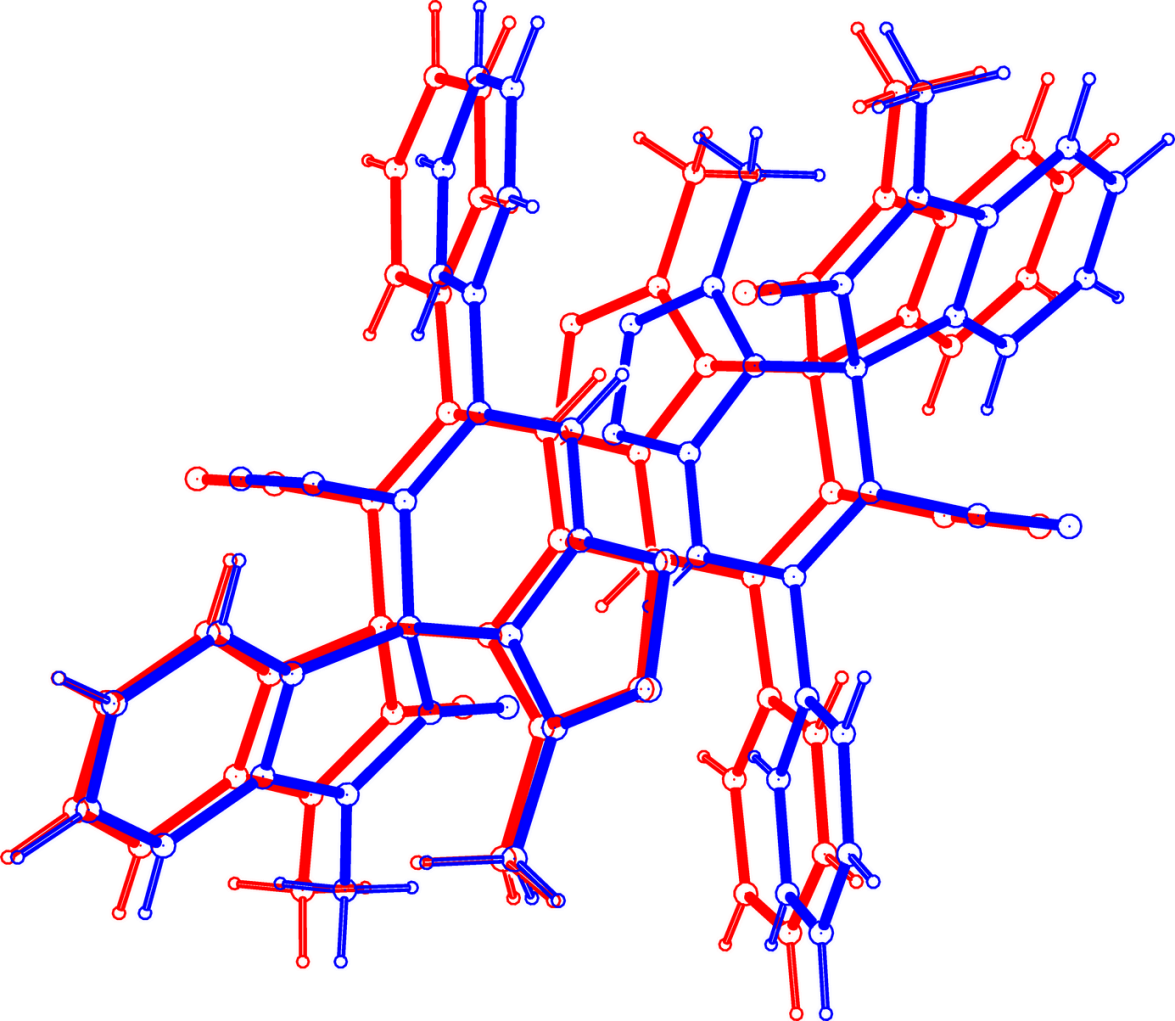
Superposition of mol­ecule *A* (red) and mol­ecule *B* (blue) for the title compound.

The title compound crystallizes in the monoclinic space group *P*2_1_/*c* with two mol­ecules (labelled *A* and *B*) in the asymmetric unit (Fig. 1 ▸). The mol­ecular structure is characterized by a spiro junction connecting the indoline core with the isoxazolo[5,4-*b*]pyridine ring system. The indoline moiety is substituted at the isatin with a phenyl group and at the 2-position with a keto group, forming a 2-oxoindoline derivative. The two mol­ecules have slightly different conformations. Fig. 2 ▸ shows a superposition of the two mol­ecules using *PLATON* (Spek *et al.*, 2020 ▸) highlighting the differences in their conformations; the root-mean-square deviation is 0.140 Å after inversion. The observed deviation is attributed to the torsional twisting of the phenyl ring with respect to the pyridine ring. For example, the torsion angle between atoms N2*A*—C10*A*—C14*A*—C19*A* is 39.9 (2)° in mol­ecule *A* and N2*B*—C10*B*—C14*B*—C19*B* is −32.1 (2)° in mol­ecule *B*. The bond lengths C21*A*—N4*A* [1.142 (3) Å] and C21*B*—N4*B* [1.148 (3) Å] confirm the triple-bond character. In mol­ecule *A*, atoms O1*A*, C20*A* and C22*A* deviate by −0.081 (2), 0.107 (3) and −0.244 (3) Å, respectively, from the least-squares plane through the ring to which they are attached (C1*A*–C2*A*–C7*A*–N1*A*–C8*A* and C13*A*–C12A–N3*A*–O2*A*–C11*A*). The deviations for the corresponding atoms in mol­ecule *B* are 0.059 (3), −0.076 (6) and 0.177 (3) Å, respectively. The pyridine (C11*A*–N2*A*–C10*A*–C9*A*–C1*A*–C13*A*) and phenyl (C14*A*–C19*A*) rings subtend a dihedral angle of 41.13 (10)° in mol­ecule *A* and 33.36 (10)° in mol­ecule *B*.

**Figure 3:**
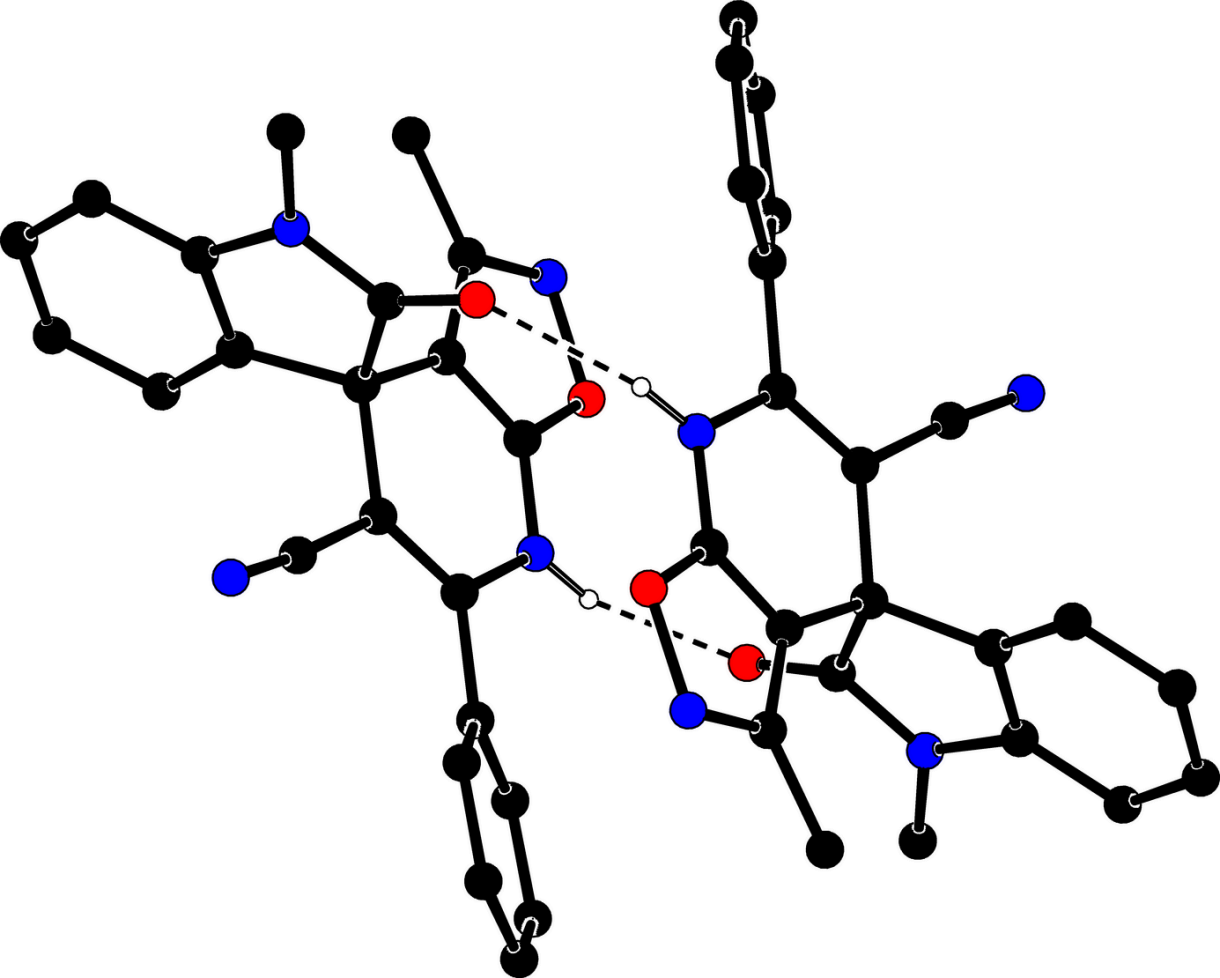
A partial packing diagram for the title compound. N—H⋯O hydrogen bonds are shown as dashed lines. H atoms not involved in these inter­actions have been omitted for clarity.

In the crystal, mol­ecules are linked by inter­molecular N—H⋯O, C—H⋯O and C—H⋯N inter­actions, forming dimers and layers parallel to the (100) plane (Table 1 ▸; Figs. 3 ▸ and 4 ▸). In addition, π–π [*Cg*1⋯*Cg*12 = 3.8057 (12) Å; *Cg*1 and *Cg*12 are the centroids of isoxazole O*2A*–N3*A*–C12*A*–C13*A*–C11*A* and phenyl C14*B*–C19*B* ring, respectively] and C—H⋯π inter­actions connect the mol­ecules within the layers that are also connected by van der Waals inter­actions.

## Synthesis and crystallization

In a 50 ml round-bottom flask, 1*H*-indole-2,3-dione (0.5 mmol) was dissolved in toluene (5 ml) and then 3-oxo-3-phenyl­propane­nitrile (0.5 mmol) and 5-amino-3-methyl­isoxazole (0.5 mmol) were added to it. To the stirring reaction mixture was added *p*-toluene­sulfonic acid (30 mole %), and stirring was continued under reflux conditions for 10 h at 383 K. The purified compound, obtained by column chromatography, was crystallized from ethanol solution.

## Refinement

Crystal data, data collection and structure refinement details are summarized in Table 2 ▸. Additional electron density was localized in voids (398 Å^3^ solvent accessible volume) summing up to 92 electrons, which corresponds to approximately 3.5 mol­ecules of ethanol. 

## Supplementary Material

Crystal structure: contains datablock(s) global, I. DOI: 10.1107/S2414314626002324/vm4076sup1.cif

Structure factors: contains datablock(s) I. DOI: 10.1107/S2414314626002324/vm4076Isup2.hkl

Supporting information file. DOI: 10.1107/S2414314626002324/vm4076Isup3.cml

CCDC references: 1983983, 2534815

Additional supporting information:  crystallographic information; 3D view; checkCIF report

## Figures and Tables

**Figure 4:**
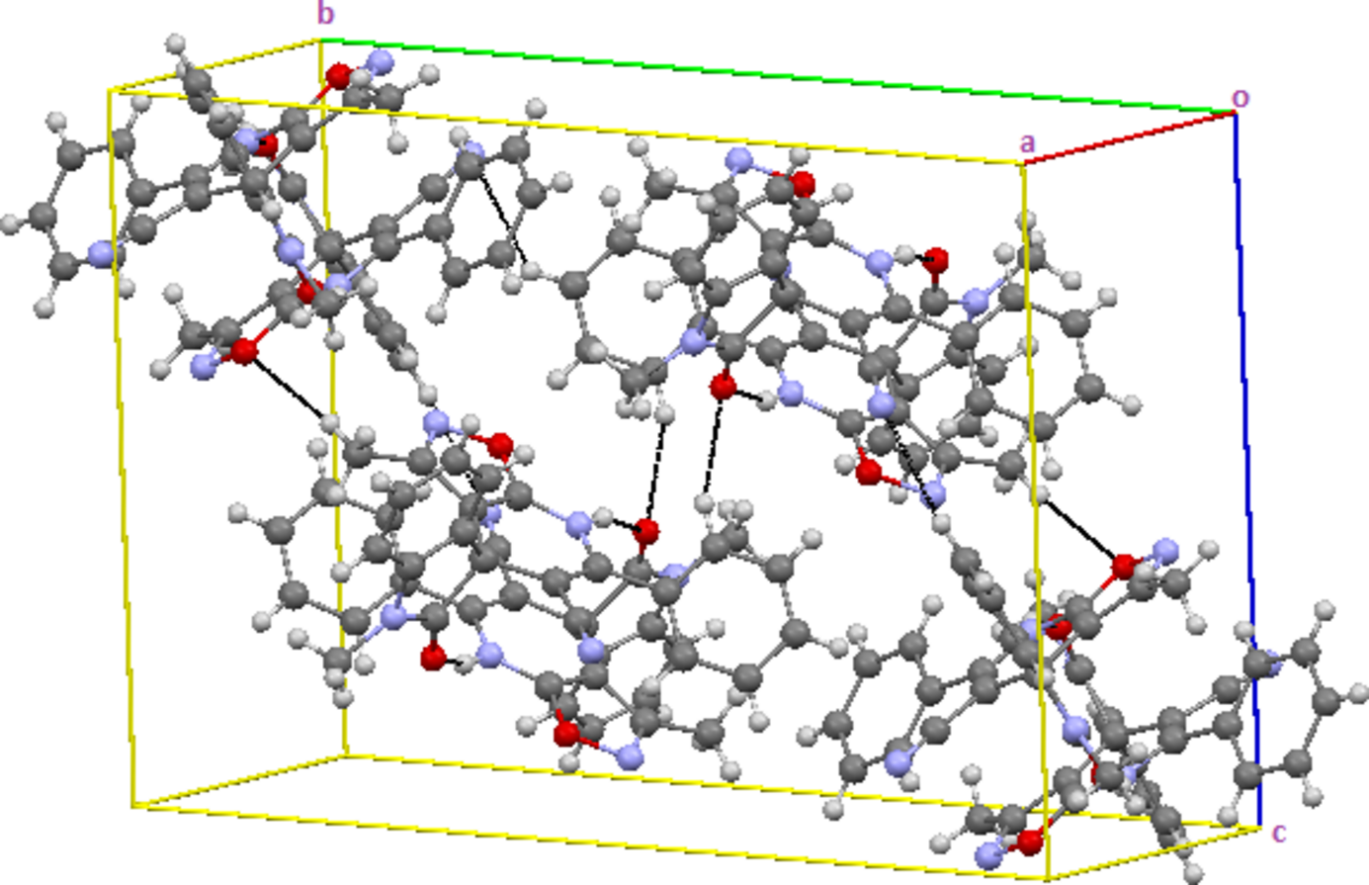
A view of the mol­ecular packing showing the C—H⋯O and C—H⋯N inter­actions.

**Table 1:** Hydrogen-bond geometry (Å, °) *Cg*2 is the centroid of the N1*A*/C7*A*/C2*A*/C1*A*/C8*A* ring.

*D*—H⋯*A*	*D*—H	H⋯*A*	*D*⋯*A*	*D*—H⋯*A*
N2*A*—H2*A*⋯O1*B*	0.86	2.09	2.910 (2)	159
N2*B*—H2*B*⋯O1*A*	0.86	1.92	2.731 (2)	156
C6*A*—H6*A*⋯N4*B*^i^	0.93	2.62	3.299 (3)	130
C6*B*—H6*B*⋯N4*A*^ii^	0.93	2.62	3.496 (3)	157
C19*A*—H19*A*⋯O1*B*^iii^	0.93	2.50	3.377 (3)	157
C22*A*—H22*B*⋯*Cg*2	0.96	2.95	3.314 (3)	103

Symmetry codes: (i) 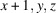; (ii) 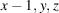; (iii) 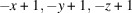.

**Table 2:** Experimental details

Crystal data	
Chemical formula	C_22_H_16_N_4_O_2_
*M*_r_	368.39
Crystal system, space group	Monoclinic, *P*2_1_/*c*
Temperature (K)	294
*a*, *b*, *c* (Å)	13.6520 (2), 19.5065 (3), 15.3599 (3)
β (°)	104.7906 (7)
*V* (Å^3^)	3954.85 (11)
*Z*	8
Radiation type	Mo *K*α
μ (mm^−1^)	0.08
Crystal size (mm)	0.28 × 0.25 × 0.16

Data collection	
Diffractometer	Bruker D8 QUEST PHOTON-100
Absorption correction	Multi-scan (*SADABS*; Krause *et al.*, 2015 ▸)
*T*_min_, *T*_max_	0.658, 0.746
No. of measured, independent and observed [*I* > 2σ(*I*)] reflections	34243, 6957, 5203
*R*_int_	0.044
(sin θ/λ)_max_ (Å^−1^)	0.595

Refinement	
*R*[*F*^2^ > 2σ(*F*^2^)], *wR*(*F*^2^), *S*	0.046, 0.132, 1.05
No. of reflections	6957
No. of parameters	509
H-atom treatment	H-atom parameters constrained
Δρ_max_, Δρ_min_ (e Å^−3^)	0.18, −0.23

Computer programs: *APEX3* and *SAINT* (Bruker, 2016 ▸), *SHELXT* (Sheldrick, 2015*a* ▸), *SHELXL2014* (Sheldrick, 2015*b* ▸) and *SHELXTL* (Sheldrick, 2008 ▸).

## full crystallographic data

### 1,3'-Dimethyl-2-oxo-6'-phenyl-7'H-spiro[indoline-3,4'-isoxazolo[5,4-b]pyridine]-5'-carbonitrile Crystal data

**Table:**

C_22_H_16_N_4_O_2_	*F*(000) = 1744
*M**_r_* = 368.39	*D*_x_ = 1.237 Mg m^−^^3^
Monoclinic, *P*2_1_/*c*	Mo *K*α radiation, λ = 0.71073 Å
*a* = 13.6520 (2) Å	Cell parameters from 9936 reflections
*b* = 19.5065 (3) Å	θ = 2.5–30.4°
*c* = 15.3599 (3) Å	µ = 0.08 mm^−^^1^
β = 104.7906 (7)°	*T* = 294 K
*V* = 3954.85 (11) Å^3^	Block, colorless
*Z* = 8	0.28 × 0.25 × 0.16 mm

### 1,3'-Dimethyl-2-oxo-6'-phenyl-7'H-spiro[indoline-3,4'-isoxazolo[5,4-b]pyridine]-5'-carbonitrile Data collection

**Table:**

Bruker D8 QUEST PHOTON-100 diffractometer	5203 reflections with *I* > 2σ(*I*)
ω and φ scans	*R*_int_ = 0.044
Absorption correction: multi-scan (SADABS; Krause *et al.*, 2015)	θ_max_ = 25.0°, θ_min_ = 2.5°
*T*_min_ = 0.658, *T*_max_ = 0.746	*h* = −16→16
34243 measured reflections	*k* = −22→23
6957 independent reflections	*l* = −18→18

### 1,3'-Dimethyl-2-oxo-6'-phenyl-7'H-spiro[indoline-3,4'-isoxazolo[5,4-b]pyridine]-5'-carbonitrile Refinement

**Table:**

Refinement on *F*^2^	0 restraints
Least-squares matrix: full	Hydrogen site location: inferred from neighbouring sites
*R*[*F*^2^ > 2σ(*F*^2^)] = 0.046	H-atom parameters constrained
*wR*(*F*^2^) = 0.132	*w* = 1/[σ^2^(*F*_o_^2^) + (0.0521*P*)^2^ + 1.8268*P*] where *P* = (*F*_o_^2^ + 2*F*_c_^2^)/3
*S* = 1.05	(Δ/σ)_max_ < 0.001
6957 reflections	Δρ_max_ = 0.18 e Å^−^^3^
509 parameters	Δρ_min_ = −0.23 e Å^−^^3^

### 1,3'-Dimethyl-2-oxo-6'-phenyl-7'H-spiro[indoline-3,4'-isoxazolo[5,4-b]pyridine]-5'-carbonitrile Special details

**Table:**

Geometry. All esds (except the esd in the dihedral angle between two l.s. planes) are estimated using the full covariance matrix. The cell esds are taken into account individually in the estimation of esds in distances, angles and torsion angles; correlations between esds in cell parameters are only used when they are defined by crystal symmetry. An approximate (isotropic) treatment of cell esds is used for estimating esds involving l.s. planes.
Refinement. Hydrogen atoms were positioned geometrically (C—H = 0.93–0.98 Å) and allowed to ride on their parent atoms, with *U*_iso_(H) = 1.5*U*_eq_(C) for methyl H and 1.2*U*_eq_ (C) for other H atoms.

### 1,3'-Dimethyl-2-oxo-6'-phenyl-7'H-spiro[indoline-3,4'-isoxazolo[5,4-b]pyridine]-5'-carbonitrile Fractional atomic coordinates and isotropic or equivalent isotropic displacement parameters (Å^2^)

**Table:**

	*x*	*y*	*z*	*U*_iso_*/*U*_eq_	
C1A	0.89488 (13)	0.63143 (9)	0.62356 (13)	0.0369 (4)	
C2A	0.98261 (14)	0.63155 (10)	0.57954 (14)	0.0429 (5)	
C3A	0.99273 (18)	0.60113 (12)	0.50136 (17)	0.0587 (6)	
H3A	0.943197	0.571606	0.468940	0.070*	
C4A	1.0791 (2)	0.61569 (14)	0.4721 (2)	0.0733 (8)	
H4A	1.087295	0.595662	0.419457	0.088*	
C5A	1.15200 (19)	0.65924 (15)	0.5200 (2)	0.0767 (8)	
H5A	1.208834	0.668357	0.499043	0.092*	
C6A	1.14306 (17)	0.68984 (13)	0.59863 (19)	0.0652 (7)	
H6A	1.192735	0.719233	0.631116	0.078*	
C7A	1.05747 (14)	0.67504 (11)	0.62696 (16)	0.0479 (5)	
C8A	0.93893 (13)	0.67726 (10)	0.70769 (14)	0.0398 (5)	
C9A	0.86335 (13)	0.56076 (9)	0.65162 (13)	0.0385 (4)	
C10A	0.76637 (13)	0.53730 (9)	0.63614 (13)	0.0366 (4)	
C11A	0.70945 (13)	0.63465 (10)	0.54961 (13)	0.0370 (4)	
C12A	0.78519 (15)	0.72100 (10)	0.50493 (14)	0.0435 (5)	
C13A	0.80136 (13)	0.66314 (9)	0.56297 (13)	0.0369 (4)	
C14A	0.73617 (13)	0.46977 (10)	0.66480 (14)	0.0399 (5)	
C15A	0.77555 (16)	0.44455 (11)	0.75064 (15)	0.0499 (5)	
H15A	0.825165	0.469124	0.791455	0.060*	
C16A	0.7413 (2)	0.38276 (13)	0.77603 (19)	0.0662 (7)	
H16A	0.766967	0.366440	0.834299	0.079*	
C17A	0.6697 (2)	0.34550 (13)	0.7155 (2)	0.0730 (8)	
H17A	0.647519	0.303735	0.732660	0.088*	
C18A	0.63098 (19)	0.36966 (13)	0.6303 (2)	0.0711 (7)	
H18A	0.583315	0.343932	0.589193	0.085*	
C19A	0.66221 (16)	0.43201 (11)	0.60502 (16)	0.0538 (6)	
H19A	0.633635	0.448947	0.547545	0.065*	
C20A	1.08696 (18)	0.75127 (14)	0.76617 (19)	0.0746 (8)	
H20A	1.080156	0.795770	0.738583	0.112*	
H20B	1.157133	0.738346	0.782933	0.112*	
H20C	1.061405	0.752560	0.818862	0.112*	
C21A	0.94644 (15)	0.51870 (11)	0.69577 (16)	0.0509 (5)	
C22A	0.85997 (18)	0.77382 (12)	0.49384 (18)	0.0626 (6)	
H22A	0.906826	0.753838	0.463961	0.094*	
H22B	0.896233	0.790473	0.551943	0.094*	
H22C	0.825004	0.811175	0.458458	0.094*	
O1A	0.89919 (10)	0.69017 (8)	0.76840 (10)	0.0520 (4)	
O2A	0.63896 (10)	0.66892 (7)	0.48864 (9)	0.0481 (4)	
N1A	1.02952 (12)	0.70132 (9)	0.70255 (12)	0.0482 (4)	
N2A	0.68608 (11)	0.57551 (8)	0.58637 (11)	0.0409 (4)	
H2A	0.624352	0.562432	0.579059	0.049*	
N3A	0.69019 (14)	0.72635 (9)	0.45955 (12)	0.0528 (5)	
N4A	1.01792 (15)	0.48861 (11)	0.72916 (19)	0.0846 (8)	
C1B	0.49623 (13)	0.61809 (9)	0.74448 (12)	0.0344 (4)	
C2B	0.41311 (14)	0.62528 (10)	0.79318 (13)	0.0382 (4)	
C3B	0.41023 (17)	0.66276 (11)	0.86851 (14)	0.0493 (5)	
H3B	0.462583	0.692614	0.894708	0.059*	
C4B	0.3270 (2)	0.65488 (14)	0.90445 (18)	0.0664 (7)	
H4B	0.323381	0.679566	0.955323	0.080*	
C5B	0.2497 (2)	0.61024 (15)	0.86443 (19)	0.0723 (8)	
H5B	0.194912	0.605280	0.889489	0.087*	
C6B	0.25150 (17)	0.57295 (13)	0.78865 (18)	0.0611 (6)	
H6B	0.198866	0.543431	0.762059	0.073*	
C7B	0.33438 (14)	0.58113 (10)	0.75380 (14)	0.0431 (5)	
C8B	0.44666 (13)	0.56811 (10)	0.66731 (13)	0.0381 (4)	
C9B	0.52764 (13)	0.68547 (9)	0.70758 (12)	0.0361 (4)	
C10B	0.62397 (13)	0.71009 (9)	0.72305 (12)	0.0350 (4)	
C11B	0.68074 (13)	0.61514 (10)	0.81534 (12)	0.0367 (4)	
C12B	0.60877 (15)	0.52833 (10)	0.86216 (14)	0.0441 (5)	
C13B	0.58972 (14)	0.58611 (9)	0.80438 (12)	0.0356 (4)	
C14B	0.65493 (14)	0.77549 (10)	0.68854 (13)	0.0387 (4)	
C15B	0.60827 (16)	0.80081 (11)	0.60372 (15)	0.0471 (5)	
H15B	0.553062	0.777648	0.567754	0.056*	
C16B	0.64272 (19)	0.86005 (12)	0.57189 (17)	0.0589 (6)	
H16B	0.610379	0.876388	0.514923	0.071*	
C17B	0.7241 (2)	0.89481 (12)	0.62362 (19)	0.0644 (7)	
H17B	0.747369	0.934438	0.601835	0.077*	
C18B	0.77107 (19)	0.87068 (12)	0.70796 (19)	0.0631 (7)	
H18B	0.826506	0.894054	0.743221	0.076*	
C19B	0.73663 (17)	0.81194 (11)	0.74084 (16)	0.0516 (5)	
H19B	0.768351	0.796623	0.798477	0.062*	
C20B	0.29114 (19)	0.49726 (14)	0.62356 (18)	0.0694 (7)	
H20D	0.325173	0.478147	0.581661	0.104*	
H20E	0.229157	0.518549	0.591115	0.104*	
H20F	0.276290	0.461463	0.661141	0.104*	
C21B	0.44443 (15)	0.72252 (11)	0.65336 (14)	0.0430 (5)	
C22B	0.53722 (19)	0.47476 (13)	0.87759 (19)	0.0695 (7)	
H22D	0.512771	0.448528	0.823510	0.104*	
H22E	0.481144	0.496265	0.893812	0.104*	
H22F	0.571655	0.444962	0.925391	0.104*	
O1B	0.48352 (10)	0.54880 (8)	0.60710 (9)	0.0486 (4)	
O2B	0.75351 (10)	0.58099 (7)	0.87432 (10)	0.0475 (4)	
N1B	0.35605 (12)	0.54822 (9)	0.67930 (11)	0.0455 (4)	
N2B	0.70321 (11)	0.67387 (8)	0.77696 (11)	0.0406 (4)	
H2B	0.764694	0.687937	0.786014	0.049*	
N3B	0.70454 (14)	0.52351 (9)	0.90502 (12)	0.0513 (4)	
N4B	0.37283 (14)	0.74794 (11)	0.60951 (15)	0.0655 (6)	

### 1,3'-Dimethyl-2-oxo-6'-phenyl-7'H-spiro[indoline-3,4'-isoxazolo[5,4-b]pyridine]-5'-carbonitrile Atomic displacement parameters (Å^2^)

**Table:**

	*U*^11^	*U*^22^	*U*^33^	*U*^12^	*U*^13^	*U*^23^
C1A	0.0285 (9)	0.0354 (10)	0.0453 (11)	−0.0014 (7)	0.0069 (8)	−0.0009 (8)
C2A	0.0338 (10)	0.0389 (11)	0.0565 (13)	0.0007 (8)	0.0126 (9)	0.0001 (10)
C3A	0.0532 (13)	0.0555 (14)	0.0709 (16)	−0.0007 (11)	0.0223 (12)	−0.0109 (12)
C4A	0.0704 (17)	0.0775 (18)	0.0851 (19)	0.0040 (14)	0.0435 (15)	−0.0159 (15)
C5A	0.0525 (14)	0.0835 (19)	0.108 (2)	−0.0050 (14)	0.0451 (15)	−0.0057 (17)
C6A	0.0411 (12)	0.0694 (16)	0.0908 (19)	−0.0110 (11)	0.0271 (12)	−0.0081 (14)
C7A	0.0335 (10)	0.0465 (12)	0.0648 (14)	−0.0018 (9)	0.0148 (10)	0.0005 (11)
C8A	0.0286 (9)	0.0391 (11)	0.0486 (12)	−0.0014 (8)	0.0037 (9)	0.0027 (9)
C9A	0.0301 (9)	0.0346 (10)	0.0482 (12)	−0.0005 (8)	0.0051 (8)	−0.0015 (9)
C10A	0.0317 (9)	0.0364 (10)	0.0402 (11)	−0.0009 (8)	0.0064 (8)	−0.0058 (8)
C11A	0.0333 (9)	0.0390 (11)	0.0369 (10)	0.0035 (8)	0.0057 (8)	−0.0055 (8)
C12A	0.0458 (11)	0.0449 (11)	0.0393 (11)	0.0026 (9)	0.0103 (9)	0.0012 (9)
C13A	0.0343 (10)	0.0362 (10)	0.0397 (11)	0.0012 (8)	0.0085 (8)	−0.0037 (8)
C14A	0.0337 (9)	0.0363 (10)	0.0505 (12)	−0.0008 (8)	0.0122 (9)	−0.0037 (9)
C15A	0.0508 (12)	0.0433 (12)	0.0544 (13)	0.0014 (10)	0.0113 (10)	−0.0011 (10)
C16A	0.0795 (17)	0.0518 (14)	0.0701 (17)	0.0085 (13)	0.0240 (14)	0.0142 (13)
C17A	0.0758 (17)	0.0464 (14)	0.101 (2)	−0.0125 (13)	0.0316 (16)	0.0081 (15)
C18A	0.0645 (15)	0.0531 (15)	0.092 (2)	−0.0251 (12)	0.0138 (14)	−0.0055 (14)
C19A	0.0465 (12)	0.0488 (13)	0.0622 (14)	−0.0123 (10)	0.0065 (10)	−0.0017 (11)
C20A	0.0539 (14)	0.0772 (18)	0.089 (2)	−0.0288 (13)	0.0109 (13)	−0.0246 (15)
C21A	0.0350 (11)	0.0425 (12)	0.0703 (15)	−0.0028 (9)	0.0045 (10)	0.0044 (11)
C22A	0.0606 (14)	0.0569 (14)	0.0689 (16)	−0.0026 (11)	0.0139 (12)	0.0203 (12)
O1A	0.0397 (7)	0.0646 (10)	0.0520 (9)	−0.0105 (7)	0.0122 (7)	−0.0117 (8)
O2A	0.0375 (7)	0.0528 (9)	0.0483 (8)	0.0044 (6)	0.0005 (6)	0.0022 (7)
N1A	0.0334 (8)	0.0510 (10)	0.0588 (11)	−0.0119 (8)	0.0090 (8)	−0.0110 (9)
N2A	0.0258 (7)	0.0408 (9)	0.0531 (10)	−0.0028 (7)	0.0047 (7)	−0.0014 (8)
N3A	0.0536 (11)	0.0528 (11)	0.0486 (11)	0.0038 (9)	0.0071 (9)	0.0089 (9)
N4A	0.0404 (11)	0.0651 (14)	0.134 (2)	0.0083 (10)	−0.0035 (12)	0.0224 (14)
C1B	0.0328 (9)	0.0350 (10)	0.0347 (10)	−0.0049 (8)	0.0076 (8)	−0.0013 (8)
C2B	0.0379 (10)	0.0367 (10)	0.0411 (11)	0.0017 (8)	0.0121 (8)	0.0062 (9)
C3B	0.0533 (12)	0.0483 (12)	0.0469 (12)	0.0046 (10)	0.0139 (10)	−0.0022 (10)
C4B	0.0740 (17)	0.0739 (17)	0.0610 (15)	0.0132 (14)	0.0350 (13)	−0.0020 (13)
C5B	0.0624 (16)	0.088 (2)	0.0810 (19)	0.0044 (14)	0.0445 (15)	0.0079 (16)
C6B	0.0477 (12)	0.0665 (15)	0.0745 (17)	−0.0093 (11)	0.0253 (12)	0.0046 (13)
C7B	0.0377 (10)	0.0453 (11)	0.0474 (12)	−0.0039 (9)	0.0131 (9)	0.0060 (9)
C8B	0.0338 (9)	0.0380 (10)	0.0405 (11)	−0.0040 (8)	0.0060 (8)	0.0019 (9)
C9B	0.0348 (9)	0.0368 (10)	0.0368 (10)	−0.0029 (8)	0.0094 (8)	0.0000 (8)
C10B	0.0376 (10)	0.0338 (10)	0.0343 (10)	−0.0045 (8)	0.0103 (8)	−0.0065 (8)
C11B	0.0354 (10)	0.0366 (10)	0.0352 (10)	0.0002 (8)	0.0036 (8)	−0.0048 (8)
C12B	0.0483 (11)	0.0399 (11)	0.0437 (11)	−0.0004 (9)	0.0114 (9)	0.0000 (9)
C13B	0.0367 (10)	0.0345 (10)	0.0351 (10)	−0.0032 (8)	0.0081 (8)	−0.0027 (8)
C14B	0.0408 (10)	0.0347 (10)	0.0442 (11)	−0.0038 (8)	0.0175 (9)	−0.0043 (9)
C15B	0.0501 (12)	0.0425 (12)	0.0517 (13)	−0.0037 (9)	0.0187 (10)	0.0018 (10)
C16B	0.0731 (15)	0.0489 (13)	0.0605 (15)	−0.0038 (12)	0.0277 (13)	0.0084 (12)
C17B	0.0822 (17)	0.0404 (13)	0.0817 (19)	−0.0144 (12)	0.0416 (15)	0.0012 (13)
C18B	0.0644 (15)	0.0472 (13)	0.0804 (19)	−0.0228 (11)	0.0237 (13)	−0.0157 (13)
C19B	0.0570 (13)	0.0436 (12)	0.0543 (13)	−0.0152 (10)	0.0146 (11)	−0.0066 (10)
C20B	0.0645 (15)	0.0754 (17)	0.0663 (16)	−0.0387 (13)	0.0130 (13)	−0.0155 (14)
C21B	0.0375 (11)	0.0442 (11)	0.0482 (12)	−0.0057 (9)	0.0125 (9)	0.0066 (10)
C22B	0.0673 (15)	0.0582 (15)	0.0818 (18)	−0.0072 (12)	0.0170 (14)	0.0254 (14)
O1B	0.0457 (8)	0.0577 (9)	0.0436 (8)	−0.0068 (7)	0.0134 (7)	−0.0110 (7)
O2B	0.0399 (7)	0.0441 (8)	0.0516 (9)	−0.0004 (6)	−0.0012 (6)	0.0001 (7)
N1B	0.0397 (9)	0.0493 (10)	0.0474 (10)	−0.0159 (8)	0.0111 (8)	−0.0071 (8)
N2B	0.0317 (8)	0.0397 (9)	0.0478 (10)	−0.0088 (7)	0.0055 (7)	−0.0015 (8)
N3B	0.0562 (11)	0.0428 (10)	0.0506 (11)	0.0008 (8)	0.0057 (9)	0.0054 (8)
N4B	0.0436 (10)	0.0741 (14)	0.0755 (14)	0.0012 (10)	0.0092 (10)	0.0245 (12)

### 1,3'-Dimethyl-2-oxo-6'-phenyl-7'H-spiro[indoline-3,4'-isoxazolo[5,4-b]pyridine]-5'-carbonitrile Geometric parameters (Å, º)

**Table:**

C1A—C13A	1.507 (3)	C1B—C13B	1.505 (3)
C1A—C2A	1.518 (3)	C1B—C2B	1.517 (3)
C1A—C9A	1.538 (3)	C1B—C9B	1.535 (2)
C1A—C8A	1.560 (3)	C1B—C8B	1.550 (3)
C2A—C3A	1.378 (3)	C2B—C3B	1.378 (3)
C2A—C7A	1.384 (3)	C2B—C7B	1.389 (3)
C3A—C4A	1.394 (3)	C3B—C4B	1.392 (3)
C3A—H3A	0.9300	C3B—H3B	0.9300
C4A—C5A	1.371 (4)	C4B—C5B	1.385 (4)
C4A—H4A	0.9300	C4B—H4B	0.9300
C5A—C6A	1.380 (4)	C5B—C6B	1.378 (4)
C5A—H5A	0.9300	C5B—H5B	0.9300
C6A—C7A	1.377 (3)	C6B—C7B	1.379 (3)
C6A—H6A	0.9300	C6B—H6B	0.9300
C7A—N1A	1.408 (3)	C7B—N1B	1.408 (3)
C8A—O1A	1.219 (2)	C8B—O1B	1.220 (2)
C8A—N1A	1.344 (2)	C8B—N1B	1.353 (2)
C9A—C10A	1.363 (2)	C9B—C10B	1.363 (2)
C9A—C21A	1.424 (3)	C9B—C21B	1.423 (3)
C10A—N2A	1.383 (2)	C10B—N2B	1.377 (2)
C10A—C14A	1.480 (3)	C10B—C14B	1.484 (3)
C11A—O2A	1.338 (2)	C11B—C13B	1.337 (3)
C11A—C13A	1.339 (3)	C11B—O2B	1.339 (2)
C11A—N2A	1.358 (2)	C11B—N2B	1.359 (2)
C12A—N3A	1.310 (3)	C12B—N3B	1.309 (3)
C12A—C13A	1.420 (3)	C12B—C13B	1.417 (3)
C12A—C22A	1.491 (3)	C12B—C22B	1.490 (3)
C14A—C15A	1.381 (3)	C14B—C15B	1.387 (3)
C14A—C19A	1.390 (3)	C14B—C19B	1.392 (3)
C15A—C16A	1.384 (3)	C15B—C16B	1.383 (3)
C15A—H15A	0.9300	C15B—H15B	0.9300
C16A—C17A	1.373 (4)	C16B—C17B	1.369 (3)
C16A—H16A	0.9300	C16B—H16B	0.9300
C17A—C18A	1.364 (4)	C17B—C18B	1.374 (4)
C17A—H17A	0.9300	C17B—H17B	0.9300
C18A—C19A	1.377 (3)	C18B—C19B	1.382 (3)
C18A—H18A	0.9300	C18B—H18B	0.9300
C19A—H19A	0.9300	C19B—H19B	0.9300
C20A—N1A	1.459 (3)	C20B—N1B	1.456 (3)
C20A—H20A	0.9600	C20B—H20D	0.9600
C20A—H20B	0.9600	C20B—H20E	0.9600
C20A—H20C	0.9600	C20B—H20F	0.9600
C21A—N4A	1.142 (3)	C21B—N4B	1.148 (3)
C22A—H22A	0.9600	C22B—H22D	0.9600
C22A—H22B	0.9600	C22B—H22E	0.9600
C22A—H22C	0.9600	C22B—H22F	0.9600
O2A—N3A	1.450 (2)	O2B—N3B	1.445 (2)
N2A—H2A	0.8600	N2B—H2B	0.8600
			
C13A—C1A—C2A	111.38 (16)	C13B—C1B—C2B	111.18 (15)
C13A—C1A—C9A	106.69 (14)	C13B—C1B—C9B	107.90 (14)
C2A—C1A—C9A	115.80 (15)	C2B—C1B—C9B	114.69 (15)
C13A—C1A—C8A	111.28 (15)	C13B—C1B—C8B	110.07 (15)
C2A—C1A—C8A	100.77 (14)	C2B—C1B—C8B	101.52 (14)
C9A—C1A—C8A	110.96 (16)	C9B—C1B—C8B	111.39 (15)
C3A—C2A—C7A	119.87 (19)	C3B—C2B—C7B	120.65 (18)
C3A—C2A—C1A	130.81 (18)	C3B—C2B—C1B	130.43 (18)
C7A—C2A—C1A	109.09 (17)	C7B—C2B—C1B	108.73 (16)
C2A—C3A—C4A	118.3 (2)	C2B—C3B—C4B	118.4 (2)
C2A—C3A—H3A	120.8	C2B—C3B—H3B	120.8
C4A—C3A—H3A	120.8	C4B—C3B—H3B	120.8
C5A—C4A—C3A	120.7 (2)	C5B—C4B—C3B	120.0 (2)
C5A—C4A—H4A	119.6	C5B—C4B—H4B	120.0
C3A—C4A—H4A	119.6	C3B—C4B—H4B	120.0
C4A—C5A—C6A	121.6 (2)	C6B—C5B—C4B	122.1 (2)
C4A—C5A—H5A	119.2	C6B—C5B—H5B	119.0
C6A—C5A—H5A	119.2	C4B—C5B—H5B	119.0
C7A—C6A—C5A	117.2 (2)	C5B—C6B—C7B	117.4 (2)
C7A—C6A—H6A	121.4	C5B—C6B—H6B	121.3
C5A—C6A—H6A	121.4	C7B—C6B—H6B	121.3
C6A—C7A—C2A	122.3 (2)	C6B—C7B—C2B	121.5 (2)
C6A—C7A—N1A	127.9 (2)	C6B—C7B—N1B	128.8 (2)
C2A—C7A—N1A	109.76 (17)	C2B—C7B—N1B	109.68 (16)
O1A—C8A—N1A	124.67 (19)	O1B—C8B—N1B	125.66 (18)
O1A—C8A—C1A	126.82 (16)	O1B—C8B—C1B	126.14 (16)
N1A—C8A—C1A	108.51 (17)	N1B—C8B—C1B	108.18 (16)
C10A—C9A—C21A	120.67 (18)	C10B—C9B—C21B	120.92 (17)
C10A—C9A—C1A	125.50 (17)	C10B—C9B—C1B	125.84 (17)
C21A—C9A—C1A	113.82 (15)	C21B—C9B—C1B	113.24 (15)
C9A—C10A—N2A	120.86 (17)	C9B—C10B—N2B	119.92 (17)
C9A—C10A—C14A	125.36 (17)	C9B—C10B—C14B	126.23 (17)
N2A—C10A—C14A	113.71 (15)	N2B—C10B—C14B	113.83 (15)
O2A—C11A—C13A	112.23 (17)	C13B—C11B—O2B	112.32 (17)
O2A—C11A—N2A	120.82 (16)	C13B—C11B—N2B	127.46 (17)
C13A—C11A—N2A	126.81 (17)	O2B—C11B—N2B	120.17 (16)
N3A—C12A—C13A	111.80 (18)	N3B—C12B—C13B	111.85 (18)
N3A—C12A—C22A	119.85 (19)	N3B—C12B—C22B	118.75 (19)
C13A—C12A—C22A	128.31 (19)	C13B—C12B—C22B	129.39 (19)
C11A—C13A—C12A	104.13 (17)	C11B—C13B—C12B	104.02 (17)
C11A—C13A—C1A	122.48 (17)	C11B—C13B—C1B	121.16 (17)
C12A—C13A—C1A	133.25 (17)	C12B—C13B—C1B	134.74 (17)
C15A—C14A—C19A	118.81 (19)	C15B—C14B—C19B	117.93 (19)
C15A—C14A—C10A	122.06 (18)	C15B—C14B—C10B	122.51 (17)
C19A—C14A—C10A	119.05 (18)	C19B—C14B—C10B	119.51 (18)
C14A—C15A—C16A	120.1 (2)	C16B—C15B—C14B	120.9 (2)
C14A—C15A—H15A	119.9	C16B—C15B—H15B	119.5
C16A—C15A—H15A	119.9	C14B—C15B—H15B	119.5
C17A—C16A—C15A	120.2 (2)	C17B—C16B—C15B	120.5 (2)
C17A—C16A—H16A	119.9	C17B—C16B—H16B	119.8
C15A—C16A—H16A	119.9	C15B—C16B—H16B	119.8
C18A—C17A—C16A	120.1 (2)	C16B—C17B—C18B	119.5 (2)
C18A—C17A—H17A	120.0	C16B—C17B—H17B	120.3
C16A—C17A—H17A	120.0	C18B—C17B—H17B	120.3
C17A—C18A—C19A	120.3 (2)	C17B—C18B—C19B	120.5 (2)
C17A—C18A—H18A	119.9	C17B—C18B—H18B	119.7
C19A—C18A—H18A	119.9	C19B—C18B—H18B	119.7
C18A—C19A—C14A	120.4 (2)	C18B—C19B—C14B	120.7 (2)
C18A—C19A—H19A	119.8	C18B—C19B—H19B	119.7
C14A—C19A—H19A	119.8	C14B—C19B—H19B	119.7
N1A—C20A—H20A	109.5	N1B—C20B—H20D	109.5
N1A—C20A—H20B	109.5	N1B—C20B—H20E	109.5
H20A—C20A—H20B	109.5	H20D—C20B—H20E	109.5
N1A—C20A—H20C	109.5	N1B—C20B—H20F	109.5
H20A—C20A—H20C	109.5	H20D—C20B—H20F	109.5
H20B—C20A—H20C	109.5	H20E—C20B—H20F	109.5
N4A—C21A—C9A	174.6 (2)	N4B—C21B—C9B	174.7 (2)
C12A—C22A—H22A	109.5	C12B—C22B—H22D	109.5
C12A—C22A—H22B	109.5	C12B—C22B—H22E	109.5
H22A—C22A—H22B	109.5	H22D—C22B—H22E	109.5
C12A—C22A—H22C	109.5	C12B—C22B—H22F	109.5
H22A—C22A—H22C	109.5	H22D—C22B—H22F	109.5
H22B—C22A—H22C	109.5	H22E—C22B—H22F	109.5
C11A—O2A—N3A	106.52 (14)	C11B—O2B—N3B	106.37 (14)
C8A—N1A—C7A	111.62 (17)	C8B—N1B—C7B	111.65 (16)
C8A—N1A—C20A	122.64 (19)	C8B—N1B—C20B	123.79 (18)
C7A—N1A—C20A	125.66 (18)	C7B—N1B—C20B	124.53 (17)
C11A—N2A—C10A	116.78 (15)	C11B—N2B—C10B	117.62 (15)
C11A—N2A—H2A	121.6	C11B—N2B—H2B	121.2
C10A—N2A—H2A	121.6	C10B—N2B—H2B	121.2
C12A—N3A—O2A	105.32 (16)	C12B—N3B—O2B	105.44 (15)
			
C13A—C1A—C2A—C3A	−61.0 (3)	C13B—C1B—C2B—C3B	62.6 (3)
C9A—C1A—C2A—C3A	61.2 (3)	C9B—C1B—C2B—C3B	−60.1 (3)
C8A—C1A—C2A—C3A	−179.1 (2)	C8B—C1B—C2B—C3B	179.7 (2)
C13A—C1A—C2A—C7A	113.40 (19)	C13B—C1B—C2B—C7B	−112.24 (18)
C9A—C1A—C2A—C7A	−124.47 (18)	C9B—C1B—C2B—C7B	125.01 (17)
C8A—C1A—C2A—C7A	−4.7 (2)	C8B—C1B—C2B—C7B	4.80 (19)
C7A—C2A—C3A—C4A	−0.3 (3)	C7B—C2B—C3B—C4B	0.5 (3)
C1A—C2A—C3A—C4A	173.5 (2)	C1B—C2B—C3B—C4B	−173.9 (2)
C2A—C3A—C4A—C5A	0.0 (4)	C2B—C3B—C4B—C5B	−0.1 (4)
C3A—C4A—C5A—C6A	0.3 (5)	C3B—C4B—C5B—C6B	−0.5 (4)
C4A—C5A—C6A—C7A	−0.2 (4)	C4B—C5B—C6B—C7B	0.6 (4)
C5A—C6A—C7A—C2A	−0.1 (4)	C5B—C6B—C7B—C2B	−0.1 (3)
C5A—C6A—C7A—N1A	−177.7 (2)	C5B—C6B—C7B—N1B	178.2 (2)
C3A—C2A—C7A—C6A	0.4 (3)	C3B—C2B—C7B—C6B	−0.4 (3)
C1A—C2A—C7A—C6A	−174.7 (2)	C1B—C2B—C7B—C6B	175.07 (19)
C3A—C2A—C7A—N1A	178.4 (2)	C3B—C2B—C7B—N1B	−179.00 (18)
C1A—C2A—C7A—N1A	3.3 (2)	C1B—C2B—C7B—N1B	−3.5 (2)
C13A—C1A—C8A—O1A	66.1 (3)	C13B—C1B—C8B—O1B	−65.3 (2)
C2A—C1A—C8A—O1A	−175.7 (2)	C2B—C1B—C8B—O1B	176.82 (19)
C9A—C1A—C8A—O1A	−52.5 (3)	C9B—C1B—C8B—O1B	54.3 (3)
C13A—C1A—C8A—N1A	−113.50 (18)	C13B—C1B—C8B—N1B	113.30 (17)
C2A—C1A—C8A—N1A	4.7 (2)	C2B—C1B—C8B—N1B	−4.54 (19)
C9A—C1A—C8A—N1A	127.86 (17)	C9B—C1B—C8B—N1B	−127.06 (17)
C13A—C1A—C9A—C10A	−9.7 (3)	C13B—C1B—C9B—C10B	2.9 (3)
C2A—C1A—C9A—C10A	−134.3 (2)	C2B—C1B—C9B—C10B	127.39 (19)
C8A—C1A—C9A—C10A	111.7 (2)	C8B—C1B—C9B—C10B	−118.0 (2)
C13A—C1A—C9A—C21A	169.23 (18)	C13B—C1B—C9B—C21B	−176.91 (16)
C2A—C1A—C9A—C21A	44.6 (2)	C2B—C1B—C9B—C21B	−52.4 (2)
C8A—C1A—C9A—C21A	−69.4 (2)	C8B—C1B—C9B—C21B	62.2 (2)
C21A—C9A—C10A—N2A	−174.26 (19)	C21B—C9B—C10B—N2B	178.96 (18)
C1A—C9A—C10A—N2A	4.6 (3)	C1B—C9B—C10B—N2B	−0.8 (3)
C21A—C9A—C10A—C14A	2.4 (3)	C21B—C9B—C10B—C14B	0.5 (3)
C1A—C9A—C10A—C14A	−178.75 (18)	C1B—C9B—C10B—C14B	−179.26 (17)
O2A—C11A—C13A—C12A	0.6 (2)	O2B—C11B—C13B—C12B	−0.2 (2)
N2A—C11A—C13A—C12A	176.20 (18)	N2B—C11B—C13B—C12B	−177.52 (18)
O2A—C11A—C13A—C1A	−175.64 (16)	O2B—C11B—C13B—C1B	176.96 (16)
N2A—C11A—C13A—C1A	0.0 (3)	N2B—C11B—C13B—C1B	−0.3 (3)
N3A—C12A—C13A—C11A	−0.4 (2)	N3B—C12B—C13B—C11B	0.4 (2)
C22A—C12A—C13A—C11A	177.4 (2)	C22B—C12B—C13B—C11B	−178.3 (2)
N3A—C12A—C13A—C1A	175.2 (2)	N3B—C12B—C13B—C1B	−176.3 (2)
C22A—C12A—C13A—C1A	−7.0 (4)	C22B—C12B—C13B—C1B	5.0 (4)
C2A—C1A—C13A—C11A	134.57 (19)	C2B—C1B—C13B—C11B	−128.85 (19)
C9A—C1A—C13A—C11A	7.3 (2)	C9B—C1B—C13B—C11B	−2.3 (2)
C8A—C1A—C13A—C11A	−113.8 (2)	C8B—C1B—C13B—C11B	119.47 (19)
C2A—C1A—C13A—C12A	−40.4 (3)	C2B—C1B—C13B—C12B	47.3 (3)
C9A—C1A—C13A—C12A	−167.6 (2)	C9B—C1B—C13B—C12B	173.9 (2)
C8A—C1A—C13A—C12A	71.2 (3)	C8B—C1B—C13B—C12B	−64.4 (3)
C9A—C10A—C14A—C15A	46.2 (3)	C9B—C10B—C14B—C15B	−36.5 (3)
N2A—C10A—C14A—C15A	−136.90 (19)	N2B—C10B—C14B—C15B	145.03 (19)
C9A—C10A—C14A—C19A	−137.0 (2)	C9B—C10B—C14B—C19B	146.4 (2)
N2A—C10A—C14A—C19A	39.9 (2)	N2B—C10B—C14B—C19B	−32.1 (2)
C19A—C14A—C15A—C16A	−0.1 (3)	C19B—C14B—C15B—C16B	0.7 (3)
C10A—C14A—C15A—C16A	176.66 (19)	C10B—C14B—C15B—C16B	−176.46 (18)
C14A—C15A—C16A—C17A	1.4 (4)	C14B—C15B—C16B—C17B	0.2 (3)
C15A—C16A—C17A—C18A	−0.8 (4)	C15B—C16B—C17B—C18B	−0.5 (4)
C16A—C17A—C18A—C19A	−1.1 (4)	C16B—C17B—C18B—C19B	−0.2 (4)
C17A—C18A—C19A—C14A	2.4 (4)	C17B—C18B—C19B—C14B	1.2 (4)
C15A—C14A—C19A—C18A	−1.8 (3)	C15B—C14B—C19B—C18B	−1.4 (3)
C10A—C14A—C19A—C18A	−178.7 (2)	C10B—C14B—C19B—C18B	175.86 (19)
C13A—C11A—O2A—N3A	−0.5 (2)	C13B—C11B—O2B—N3B	0.0 (2)
N2A—C11A—O2A—N3A	−176.43 (16)	N2B—C11B—O2B—N3B	177.55 (16)
O1A—C8A—N1A—C7A	177.3 (2)	O1B—C8B—N1B—C7B	−178.58 (19)
C1A—C8A—N1A—C7A	−3.1 (2)	C1B—C8B—N1B—C7B	2.8 (2)
O1A—C8A—N1A—C20A	−5.7 (3)	O1B—C8B—N1B—C20B	3.4 (3)
C1A—C8A—N1A—C20A	173.9 (2)	C1B—C8B—N1B—C20B	−175.2 (2)
C6A—C7A—N1A—C8A	177.8 (2)	C6B—C7B—N1B—C8B	−178.0 (2)
C2A—C7A—N1A—C8A	0.0 (3)	C2B—C7B—N1B—C8B	0.4 (2)
C6A—C7A—N1A—C20A	0.9 (4)	C6B—C7B—N1B—C20B	0.0 (4)
C2A—C7A—N1A—C20A	−177.0 (2)	C2B—C7B—N1B—C20B	178.4 (2)
O2A—C11A—N2A—C10A	168.83 (16)	C13B—C11B—N2B—C10B	2.8 (3)
C13A—C11A—N2A—C10A	−6.5 (3)	O2B—C11B—N2B—C10B	−174.33 (16)
C9A—C10A—N2A—C11A	4.0 (3)	C9B—C10B—N2B—C11B	−2.1 (3)
C14A—C10A—N2A—C11A	−173.04 (16)	C14B—C10B—N2B—C11B	176.53 (16)
C13A—C12A—N3A—O2A	0.1 (2)	C13B—C12B—N3B—O2B	−0.3 (2)
C22A—C12A—N3A—O2A	−177.89 (19)	C22B—C12B—N3B—O2B	178.51 (19)
C11A—O2A—N3A—C12A	0.2 (2)	C11B—O2B—N3B—C12B	0.2 (2)

### 1,3'-Dimethyl-2-oxo-6'-phenyl-7'H-spiro[indoline-3,4'-isoxazolo[5,4-b]pyridine]-5'-carbonitrile Hydrogen-bond geometry (Å, º)

*Cg*2 is the centroid of the N1*A*/C7*A*/C2*A*/C1*A*/C8*A* ring.

**Table:**

*D*—H···*A*	*D*—H	H···*A*	*D*···*A*	*D*—H···*A*
N2*A*—H2*A*···O1*B*	0.86	2.09	2.910 (2)	159
N2*B*—H2*B*···O1*A*	0.86	1.92	2.731 (2)	156
C6*A*—H6*A*···N4*B*^i^	0.93	2.62	3.299 (3)	130
C6*B*—H6*B*···N4*A*^ii^	0.93	2.62	3.496 (3)	157
C19*A*—H19*A*···O1*B*^iii^	0.93	2.50	3.377 (3)	157
C22*A*—H22*B*···*Cg*2	0.96	2.95	3.314 (3)	103

Symmetry codes: (i) *x*+1, *y*, *z*; (ii) *x*−1, *y*, *z*; (iii) −*x*+1, −*y*+1, −*z*+1.
